# Antimicrobial Resistance and One Health: Lessons on One Health Governance and Implementation from the Fleming Fund

**DOI:** 10.1093/cid/ciag517

**Published:** 2026-10-19

**Authors:** Syed Shahid Abbas, Rebecca Maudling, Antoine Andremont, Yu Nandar Aung, Regina Ama Banu, Manisha Bista, Roderick M Card, Eileen Chappell, Etienne Chevanne, Mauricio Coppo, Stanley Fenwick, Nigel French, William H Gaze, Joanna McKenzie, Noah Obeng-Nkrumah, Garima Singh, Desrina Sitompul, Mark Stevenson, Shawn Ting, Jaap A Wagenaar, Evelyn Wesangula, Anke Wiethoelter, Timothy Worth, Qadeer Ahsan, Winfred Christine Amia, Mathias Ayuk Besong, Eric Sampane Donkor, James Fairfax, Heidi Hopkins, Easrat Jahan, Anis Karuniawati, Toby Leslie, Ayodele Majekodunmi, Arshnee Moodley, Athina Papadopoulou, Shahriar Rizvi, Tim Shorten, Steven Uggowitzer, Tom Weaver, Muhammad Usman Zaheer

**Affiliations:** Institute of Development Studies, University of Sussex, Falmer, Brighton, United Kingdom; Itad Ltd, Brighton, United Kingdom; Itad Ltd, Brighton, United Kingdom; One Health Consultant, Portsmouth, United Kingdom; Université Paris Cité, Paris, France; Mott MacDonald, Bangkok, Thailand; Council for Scientific and Industrial Research (CSIR)—Water Research Institute, Accra, Ghana; Mott MacDonald, Delhi, India; Animal & Plant Health Agency, Addlestone, United Kingdom; Mott MacDonald, London, United Kingdom; Mott MacDonald, Bangkok, Thailand; University of Melbourne, Melbourne, Australia; Mott MacDonald, Bangkok, Thailand; College of Environmental and Life Sciences, Murdoch University, Perth, Australia; Massey University, Palmerston North, New Zealand; University of Exeter, Exeter, United Kingdom; Massey University, Palmerston North, New Zealand; School of Biomedical and Allied Health Sciences, University of Ghana, Accra, Ghana; Mott MacDonald, Delhi, India; DAI, Jakarta, Indonesia; University of Melbourne, Melbourne, Australia; Menzies School of Health Research, Darwin, Australia; Division of Infectious Diseases and Immunology, Faculty of Veterinary Medicine, Utrecht University, Utrecht, The Netherlands; Itad Ltd, Brighton, United Kingdom; East, Central and Southern Africa Health Community, Arusha, Tanzania; University of Melbourne, Melbourne, Australia; Mott MacDonald, London, United Kingdom; DAI, Islamabad, Pakistan; Mott MacDonald, Kampala, Uganda; Mott MacDonald, Accra, Ghana; Department of Medical Microbiology, University of Ghana Medical School, Accra, Ghana; Mott MacDonald, London, United Kingdom; Mott MacDonald, London, United Kingdom; DAI, Dhaka, Bangladesh; Department of Microbiology, Faculty of Medicine, Universitas Indonesia, Jakarta, Indonesia; Mott MacDonald, London, United Kingdom; Ajisefini Consulting, Abuja, Nigeria; International Livestock Research Institute, Nairobi, Kenya; University of Copenhagen, Frederiksberg, Denmark; Centre for Environment, Fisheries and Aquaculture Science (CEFAS), Weymouth, United Kingdom; DAI, Dhaka, Bangladesh; Itad Ltd, Brighton, United Kingdom; Independent Consultant, Redgrave, United Kingdom; Member, Fleming Fund Expert Advisory Group, Geneva, Switzerland; DAI, Jakarta, Indonesia; Regional Office for Asia and the Pacific, Food and Agriculture Organization of the United Nations, Bangkok, Thailand

**Keywords:** One Health, antimicrobial resistance, evaluation, integration, surveillance, policy

## Abstract

This paper reflects upon the experience of the Fleming Fund in designing and implementing One Health antimicrobial resistance (AMR) programs in 25 countries and identifies practical lessons for the design of future One Health programs. The Fleming Fund experience highlights key priorities for implementing One Health effectively within AMR programs–though many of the insights should be applicable to other contexts as well, such as emerging infectious diseases and health security. One Health should be treated as an approach to problem framing and action rather than a program output, with flexibility for national adaptation. Meaningful implementation requires sustained investment beyond human health, particularly in animal and environmental sectors, to avoid symbolic engagement. Surveillance systems should consider both the role of the sector and sector-specific needs, supported by capacity for joint interpretation and use of evidence. The costs of multisectoral coordination should be explicitly resourced and both formal governance mechanisms and informal professional networks recognized as essential for building trust, sustaining collaboration, and translating data into policy-relevant action.

A One Health approach highlights the interdependence of the health of animals, humans, plants, and the environment [1]. It was developed in response to risks of emerging zoonoses and has since then been proposed as a framework for addressing global health and sustainability challenges, including antimicrobial resistance (AMR) [2, 3]. However, despite this broad consensus on the need for the approach, many challenges remain with operationalization of One Health [4], as a result of siloed ways of working, imbalances in resources, issues of professional bias and status, and limited cross-disciplinary understanding of differing sectoral objectives, incentives, and challenges [5].

The Fleming Fund was an early example of a large, international global health program underpinned by One Health principles to address AMR at global, regional, and national levels in 25 countries through a broad portfolio of activities and collaborations [6]. These targeted multilevel investments in AMR have resulted in technical and infrastructural capacity strengthening as well as accumulation of practical insights that have informed discussions on One Health globally, such as on the need for local adaptation [7].

In this paper, we synthesize the cumulative experiences of individuals associated with designing and implementing One Health approaches within the Fleming Fund program to identify practical lessons for shaping future national and global One Health initiatives. We first describe our methods and provide an overview of how One Health was conceptualized and implemented within the Fleming Fund. We then place this vision of the Fleming Fund in the context of broader discussions around One Health to identify the key contributions of Fleming Fund experience to the One Health discourse. We have organized the discussion around two key messages that represent the major conceptual and practical lessons from the Fleming Fund experience of implementing a large, multicountry One Health program. We illustrate our key messages with case studies that provide practical examples of One Health action.

## METHODS

This paper forms part of a practice-informed series synthesizing learning from Fleming Fund-supported AMR surveillance strengthening activities. This paper was developed through a narrative synthesis of the cumulative experiences of individuals closely involved with the design, implementation, and interpretation of Fleming Fund activities related to antimicrobial resistance (AMR) and One Health. Contributors had engaged with the program in different capacities at national, regional, and global levels, including as program managers, technical experts, implementers, and evaluators. The purpose was not to provide a summative evaluation of Fleming Fund performance on One Health but to document and interpret practical knowledge generated through its implementation across diverse country and institutional contexts.

The approach was designed to harvest tacit knowledge: the forms of experiential understanding that develop through practice, negotiation, and problem solving and which are often lost when programs close or staff move on [8]. Reflection on practice, structured discussion, and dialogic exchange were therefore central to the method. Accordingly, we treat the insider knowledge of authors as central inputs to the design of the paper and not as a source of “bias,” that needs to be filtered out.

The synthesis was developed through a multistage collaborative process involving structured group calls, email exchanges, and iterative inputs into shared versions of the manuscript. Forty-nine individuals were invited to contribute, of whom 34 chose to participate. At the first writing group meeting on 5 December 2025, participants agreed on the scope of the paper and confirmed interest in contributing. The group defined three objectives: to reflect on the Fleming Fund experience in designing and implementing One Health programming over multiple years across more than 25 countries, to examine specific examples of One Health partnerships, and to identify practical lessons for future national and global One Health programs.

At a second meeting on 21 January 2026, the writing group reviewed the outline, agreed on the structure, and identified key messages. Members allocated themselves to subgroups to draft sections and case studies. These messages drew on findings from previous learning products produced within the Fleming Fund, such as a literature review on One Health governance, the summative evaluation report, and a lessons learned document [7, 9, 10]. Drafts were reviewed collectively at meetings on 3 March and 9 April 2026, before being refined through further collaborative editing. All members of the writing group were invited to contribute to each version of the outline and the three drafts of the manuscript.

While there was broad agreement on both the overarching key messages, described later in the paper, there were considerable discussions on how to present arguments and substantiate them through evidence. In case of any conflicts (which evidence to present, how to build an argument), we discussed these within the Writing Group virtual meetings and email threads to resolve them collaboratively to the extent possible. This, therefore, is not a comprehensive review of what worked and what didn't but a synthesis of collective knowledge regarding key lessons. More details on the Methods for this series can be found in the series overview paper [6].

### Fleming Fund's Approach to One Health

As described in our companion paper [6], Fleming Fund adopted One Health as a “core principle” from the outset. The program was launched in 2017, a time when global guidance on how to operationalize One Health approaches for AMR was limited (Figure 1). This allowed the program to foster different models of One Health partnerships and to generate practical lessons for operationalizing One Health, as described in this paper.

**Figure 1.**
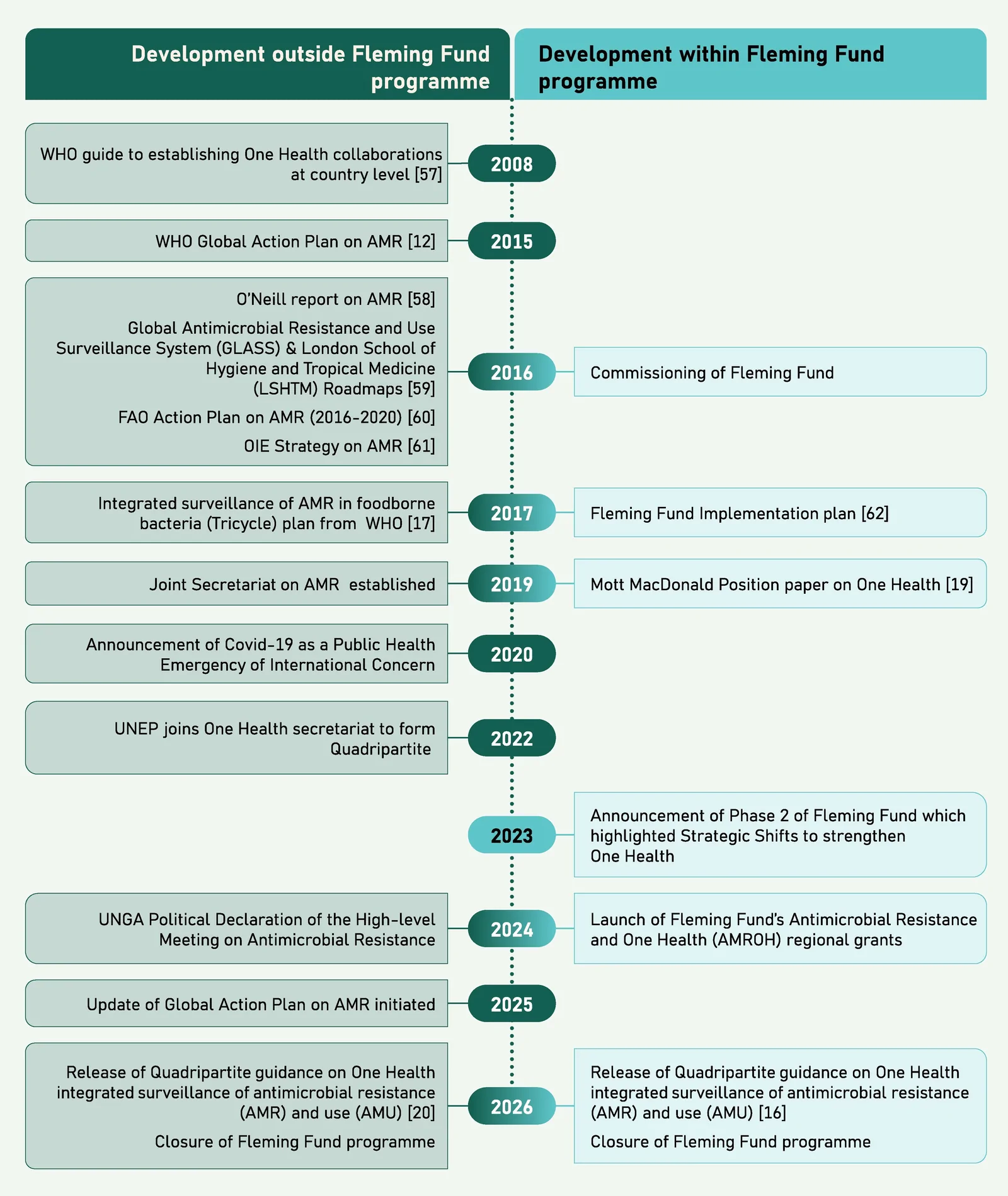
Key AMR and One Health policy milestones. Abbreviations: Covid-19, coronavirus disease 2019.

Following WHO's GLASS guidance, the Fund's initial focus was to strengthen hospital laboratory capacities for clinical AMR surveillance of priority pathogen-drug combinations. In animals, the initial focus was AMR surveillance for bacteria of public health concern in healthy poultry and additionally included monitoring antimicrobial use (AMU) in livestock because of concerns about its impact on AMR in humans. Activities in Phase 2 expanded surveillance to environment, aquaculture, and food safety in selected countries and encouraged broader participation in the WHO tricycle program, which promoted a modular approach to integrated surveillance (Box 1) [11]. Phase 2 also saw the launch of regional AMROH grants to strengthen capacities in the animal health, aquaculture, environment, and food sectors enabling a greater recognition of sector-specific priorities and constraints in national plans. Further, Phase 2 emphasized antimicrobial stewardship and practitioner engagement to promote responsible antimicrobial use across humans and animals including aquatic species.

Box 1. **Case Study 1: Integrated Surveillance—Experiences From Conducting Tricycle in Indonesia and Ghana**

The Tricycle protocol, developed by the WHO-Advisory Group on Integrated Surveillance of Antimicrobial Resistance, provides a simplified, standardized methodology to establish multisectoral integrated surveillance [40]. It is designed to detect extended-spectrum, beta-lactamase producing *Escherichia coli* (ESBL-Ec) in humans (both hospital and community settings), food producing animals (poultry), and the environment. Although its primary outcomes describe the presence and distribution of ESBL-Ec across sectors, several countries have found the sequencing and molecular characterization of resistance genes to be also informative. In countries such as Ghana, this has enabled identification of shared ESBL types across sectors, enhancing understanding of the occurrence and dissemination of resistance [41].

Therefore, Tricycle shows the value of integrated surveillance, although additional studies are needed to elucidate the size and direction of transmission. There are currently at least 22 countries worldwide that have carried out one or more Tricycle studies [38]. The Fleming Fund adopted the Tricycle protocol and facilitated its implementation through country grants, the Fleming Fellowship scheme, and other funding mechanisms. Indonesia (Jakarta) and Ghana (Greater Accra Region) implemented Tricycle in 2017–2021 [41–43]

In Indonesia, early Tricycle findings indicated increasing levels of ESBL-Ec in broiler chicken surveillance [44]. In response, a second round of sampling expanded the original protocol to include ESBL-producing *Klebsiella pneumoniae*, reflecting concerns in the human health sector. This adapted approach, termed E-Trike, was implemented collaboratively by Fleming Fellows, illustrating problem-driven expansion of integrated surveillance [45].

In Ghana, genomic integration linked Tricycle activities with the national sequencing hub, demonstrating how a single high-capacity facility can support multisectoral AMR investigations. During the second phase of Tricycle (2023–25), a multisectoral TWG, drawn from the national AMR surveillance program, was established to steer the project. A composite WHONET® database integrating human, animal, and environment modules enabled cross-sectoral data comparison and reporting to GLASS. In addition, a centralized biorepository was established to support future genomic analysis.

Across countries, common challenges included procurement of reagents (in early phases of Tricycle before support from Fleming Fund came in), limited microbiological capacity in the environmental sector, and difficulties integrating data across sectors with uneven laboratory capabilities. In conclusion, in both Indonesia and Ghana, Tricycle generated valuable surveillance outputs and fostered new institutional collaborations across sectors that had previously worked largely in isolation [38].

Fleming Fund's approach to incorporating surveillance into a decision-making framework was informed by the Global Action Plan on AMR, involving national program leadership through multisectoral AMR Coordination Committees. Sharing and analysis of surveillance data across sectors would take place through Technical Working Groups (TWGs), which would recommend both sectoral-based and multisectoral policies and programs to the multisectoral AMR Coordination Committee [12].

The Fleming Fund work plan for each country was developed through a consultative process that involved review of governance structures, assessment of infrastructure, and technical capacities relevant to the implementation of AMR National Action Plans and political engagement to confirm feasibility and local appetite amidst national priorities. Programs were co-developed with country teams and approved by the head of the National AMR committee. At the start of the Fleming Fund program, raising awareness and understanding of AMR within the sectors and potential associations between the sectors was identified as a key priority, which helped guide the design of the program in the different countries.

The Fleming Fund developed a gradient-based approach to implementing a One Health surveillance and decision-making system (Figure 2). Recognizing that countries would engage with One Health principles in different ways and at different rates of progress, the program aimed to provide tailored support to each of the sectors in Fleming-supported countries at different stages of the program [13]. However, as discussed in the evaluation report [7] and also captured in the two key messages later in the paper, the program encountered several hurdles in operationalizing this version of a One Health approach.

**Figure 2.**
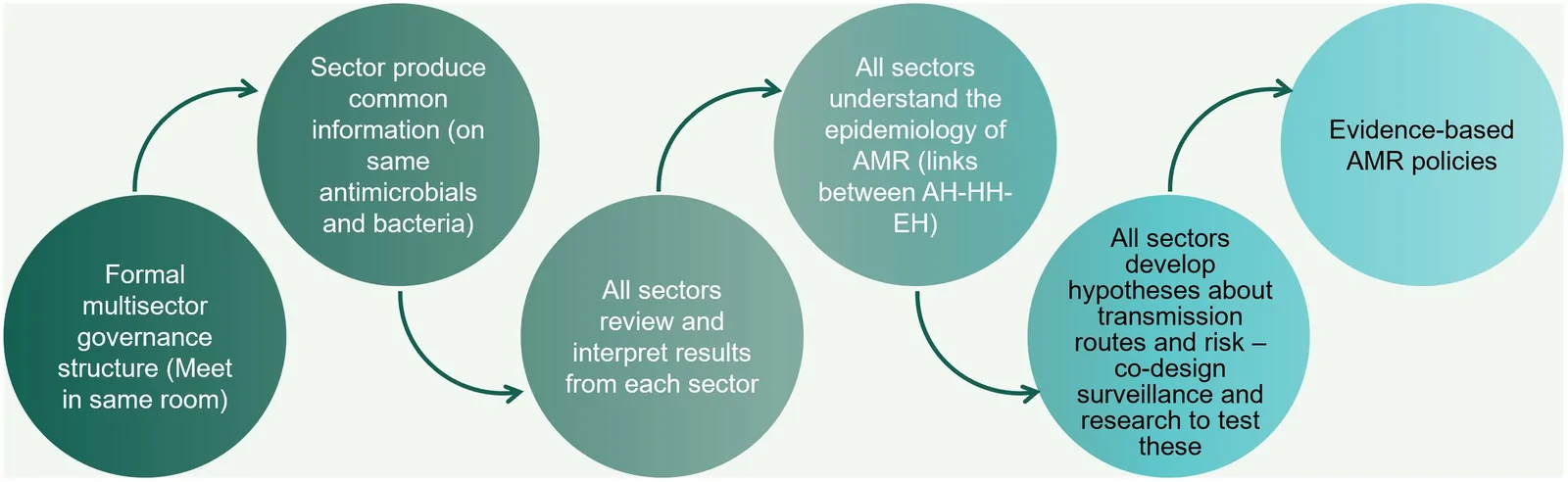
Gradient-based approach to One Health in Fleming Fund. Abbreviations: AH, animal health; AMR, antimicrobial resistance; EH, environmental health; HH, human health.

Fleming Fund faced major disruptions to its planned activities from the COVID-19 pandemic. However, it was able to leverage its investments in improving laboratories and biosafety training to strengthen national pandemic responses as well as to support collaborations across sectors through the Fellowship program and through shared resourcing (see Boxes 2 and 4). The Fleming Fellowship program was established to support the professional development of human and animal health practitioners and policymakers in Fleming Fund-supported countries. Fellows were typically mentored by an expert outside their countries and were encouraged to undertake collaborative research and policy projects with their counterparts from other sectors, as described in Box 2.

Box 2. **Case Study 2: One Health Collaborations in the Fleming Fellowship Program**

The Fleming Fund Fellowship scheme aimed to support the professional development of AMR professionals in Fleming fund–supported countries, leading to sustainable, context-relevant capacity development while strengthening national AMR responses. It built a global community of researchers, practitioners, and policymakers able to contribute to AMR awareness and reduction.

Implemented in two phases, the scheme supported nearly 400 Fellows across 20 countries, drawn from human health, animal health (including aquaculture), and environmental health backgrounds. Selection considered technical expertise, leadership potential, and ability to influence systems. Professional Fellowships targeted early and mid-career staff while Policy Fellowships supported senior staff involved in policy development. Structured mentoring with host institutions built cross-sector consortia, supporting One Health alignment across their cohorts.

The scheme faced operational challenges, particularly COVID-19 and seasonal animal disease outbreaks, which required Fellows to lead response activities, limiting their availability. However, these disruptions also created opportunities: animal health fellows and laboratories supported national response, strengthening skills and cross-sector collaborations. Despite disruptions, the Fellowship contributed to AMR surveillance, multisector collaboration, and policy-relevant evidence.

In Nigeria, Fellows helped establish a One Health community of practice beyond formal AMR coordination structures. Fellowship-supported research generated evidence of AMR transmission among slaughterhouse workers, cattle meat, and surrounding environments and informed national discussions on surveillance priorities, biosecurity, and integrated interventions [46, 47]. Similar outcomes were observed in Sri Lanka, where fellows studied AMR-related knowledge, attitudes, and practices among One Health stakeholders [48]. In Ghana, fellows documented unsupervised use of critically important antibiotics in communities and poultry production, later convening the country's first national One Health AMR conference [49, 50].

An enduring outcome of the scheme is its network of 357 alumni, generating ongoing professional collaborations and personal networks. At a regional meeting in March 2022, fellows from Kenya, Malawi, Uganda, and Zambia highlighted improved understanding across sectors, increased collaboration, and collective approaches to harmonization while recognizing contextual differences across the four countries (Chappell pers comm 10th April 2022).

Overall, the Fellowship scheme demonstrated the value of investing in professional development networks and multisector collaboration to strengthen national AMR responses. It supported individuals who continue to influence policy, practice, and collaboration in their countries. The Fleming Fellowship program will continue under the Fleming Initiative.

The experience of engaging closely with multiple sectors in 25 countries across Africa and Asia helped identify a wide range of challenges related to strengthening a One Health approach. These include the considerable variation in resourcing and capacity as well as the different approaches to surveillance in the different sectors. Differences in levels of commitment to collaboration, sectoral objectives, and incentive structures shape how sectors engage with One Health approaches, with implications for ownership of programs primarily focused on human health outcomes. The Fleming Fund focused on human health outcomes using a multisector approach and encountered many of the above challenges, which we elaborate upon in Message 2, below.

To summarize, the Fleming Fund adopted One Health as a core principle from the beginning even as the international guidance on One Health implementation was still being developed. Fleming Fund tailored its support within an agreed adaptive framework to meet the needs of different countries’ institutional priorities as well as to respond to different sectoral mandates and capacities. Fleming Fund's One Health approach evolved over time to include specific regional grants focused on strengthening animal health and bringing environment surveillance and action within its ambit. As we describe later in the paper, there is evidence of ongoing progress in multisector engagement across the Fleming Fund countries, even though challenges remain.

In this paper, we identify several insights from the Fleming Fund experience that are not currently well reflected in One Health policy discussions. These sets of insights relate both to the external aspects of how One Health programs have been structured and organized in different programmatic contexts (Message 1) and to the internal contestations and dynamics among different One Health stakeholders that inevitably exist and influence the nature of final outcomes (Message 2). Both sets of insights are explained in the following sections.

## MESSAGE 1: ONE HEALTH COMES IN DIFFERENT FORMS

Ideas on using a One Health approach for AMR governance have evolved significantly since the Global Action Plan on AMR (2015). A 2025 review identified more than 50 national-level One Health toolkits, though only eight focused specifically on AMR [14]. Recent program guidance emphasizes the need to adapt international One Health frameworks to national institutional and technical contexts [15, 16]. However, concerns remain that many tools insufficiently address the social and political dynamics shaping multisector programs or provide limited support for managing complexity at national and subnational levels [4, 17]. In this respect, the Fleming Fund experience of implementing a One Health program in 25 countries offers practical insights into context-specific adaptation.

Across the Fleming Fund countries, AMR coordinating committees (AMRCCs) shared common features, including multisector membership, defined terms of reference, and technical substructures, yet differed markedly in leadership arrangements, decision-making authority, financing, and the balance between sectoral and shared interests. In this section, we examine the different ways in which Fleming Fund-supported countries chose to organize their One Health functions, using an operational framework on One Health to structure the section around *starting conditions*, *structure*, and *characteristics* of One Health partnerships [17].

### Starting Conditions

Evidence suggests that prior collaboration influences the formation and function of multisector partnerships [18, 19]. In several countries, One Health arrangements initially developed to respond to zoonotic disease outbreaks were later extended to AMR. For example, in Lao PDR the National Avian and Human Influenza Coordinating Office, established to address highly pathogenic avian influenza in 2004, transformed into the National Emerging Infectious Disease Coordinating Office in 2009. A similar trajectory occurred in Vietnam, where the Partnership for Avian and Human Influenza prevention became the One Health Partnership in 2016.

### Structure

#### Leadership

Countries choose different leadership models that best suit their respective institutional bureaucracies. For instance, in Senegal, multisector coordination is overseen by the Prime Minister's office through the National High Council for Global Health Security (*Haut Conseil national de la sécurité sanitaire mondiale*) [20]. In contrast, Indonesia deploys a “Coordinating Ministry” to facilitate partnerships across ministries, which have their own indicators and budgetary allocations [21, 22]. In many countries, however, AMR activities remain largely sector-led, with formal coordination mechanisms such as an AMRCC only partially operational [7]. Some countries, such as Papua New Guinea, have adopted rotating leadership to encourage joint ownership. Most countries, however, house multisector committees within health agencies, recognizing the human health emphasis of AMR. Others, like Kenya, present a hybrid model where the AMRCC is primarily hosted within the Kenya National Public Health Institute, while the chairing rotates between ministries [23, 24].

#### Sub-national Devolution

While international program guidance rightly focuses on national action plans, their operationalization takes place at the local level. Larger countries with devolved administration, such as India, Pakistan, and Kenya, replicate national coordination mechanisms at provincial or county levels, such as provincial AMR subnational standing committees in Pakistan [10, 23, 25–27]. In Kenya, while policy formation occurs nationally, 47 different county governments bear responsibility for implementation. Establishing county AMR committees has been slow, highlighting the difficulty of ensuring alignment and accountability across levels [24, 28].

#### Coordination Costs

Establishing and operationalizing multisector One Health partnerships are not cost-neutral and require substantial investments for personnel and surveillance activities and stewardship. Unfortunately, in many countries, the activities of One Health coordination are funded through external agencies or without earmarked budgets. This presents a challenge in ensuring their effectiveness and sustainability [10, 15].

### Characteristics

#### Role of Informal Partnerships

Much of the programmatic guidance on One Health governance prioritizes formal coordination mechanisms between government ministries and technical agencies. However, experience from the Fleming Fund demonstrates that informal networks can play an important and a complementary role to these formal institutional coordination mechanisms [7, 9]. Even in countries with strong government institutions, such as Kenya, Zambia, and Pakistan, technical deliberative bodies such as the AMRCCs and TWGs often invite participation from technical experts, development partners, and civil society stakeholders, when needed. As described in Box 3, Bangladesh exemplifies a hybrid model combining formal government committee deliberations with multisector interactions within influential professional networks, such as One Health Bangladesh, that pre-dated state structures and facilitated policy uptake. The Fleming Fund enabled One Health exchanges through other forms of informal interactions as well. A good example is the Fellowship scheme, which resulted in cross-sector research and policy collaborations and seeded national and regional networks of AMR champions (Box 2).

Box 3. **Case Study 3: Multisector Governance in Bangladesh**

Bangladesh has progressively operationalized a One Health approach to AMR through incremental institutional development. Instead of adopting a single predefined model, policy actors have assembled a One Health architecture that links formal state institutions with professional and civil society networks. This configuration reflects a pragmatic effort to translate international One Health guidance to national administrative and political contexts.

Civil society mobilization was central to this process. One Health Bangladesh emerged in 2008 as a professional platform convening researchers, practitioners, and policy actors across human, animal, and environmental health domains. Sustained advocacy and cross-disciplinary dialogue supported the development of a National One Health Strategic Framework and Action Plan, later endorsed by the government. Thus, non-state actors played a key role in agenda setting and early institutional design, creating conditions for incorporation of One Health principles into formal governance [51].

AMR governance is now organized through interconnected national bodies under a One Health framing. The Inter-ministerial National Steering Committee provides strategic oversight across relevant ministries, while the National Technical Committee and sector-specific Technical Working Groups support implementation of the National Action Plan. This structure helps retain sectoral expertise while aligning action through a central coordinating mechanism.

Recent policy developments indicate continued consolidation. Bangladesh endorsed a revised, costed National Strategy and NAP for AMR (2023–2028) and refreshed its One Health Strategic Framework (2025–2030). Proposed adjustments include rotating leadership across core ministries, establishing a dedicated One Health Secretariat, and developing shared data tools such as a One Health AMR dashboard.

These arrangements are the product of sustained advocacy and network-building over two decades. Institutional features that supported One Health include senior political endorsement, defined committee roles, and investments in integrated surveillance protocols. However, implementation remains constrained by uneven multisectoral participation, challenges integrating surveillance systems, pressures on subnational delivery, workforce limitations, and unstable financing for environmental AMR activities [51, 52].

Overall, Bangladesh's experience shows that operationalizing One Health for AMR is an incremental governance process dependent on sustained links between state institutions and professional civil society engagement, with persistent gaps between institutional ambition and operational delivery.

#### Strength of Partnership

Organizing regular meetings and activities of multisector partnerships involves transaction costs of time and resources, which make these difficult to sustain in many countries. When coupled with histories of institutional conflicts and disciplinary hierarchies, we observed that in several countries it was more difficult to share data between national ministries than it was to share with international sectoral bodies (WHO, FAO, or WOAH). Therefore, many countries chose to restrict the scope of One Health to exchanging information or joint planning—without extending it to collaborative interventions [7, 29].

Across Fleming Fund countries, experience shows that there is no single template for applying One Health to AMR. Effective governance depends on context-specific, adaptive arrangements shaped by national institutions and politics. Multiple models can coexist, supported by leadership innovation, adequate investments in coordination infrastructure, and strong informal and professional networks that complement formal One Health structures and sustain multisector collaboration over time.

## MESSAGE 2: SECTORAL REPRESENTATION, OWNERSHIP, AND COMMITMENT TO ONE HEALTH

Fleming Fund's experience with One Health approaches offers important lessons on how multisectoral collaboration in AMR can be structured to support ownership, relevance, and sustained commitment across human, animal, and environmental systems. Its adoption of a One Health approach illustrates both the potential as well as the practical challenges of establishing multisectoral ownership for AMR surveillance and stewardship. Multisector partnerships arise when individual sectors lack the resources or knowledge to address complex problems alone [9, 30, 31]. However, experience from the Fleming Fund program underlines that without clear conceptualization, sector-specific objectives, and adequate resourcing of all sectors, One Health approaches risk weakening ownership, distorting incentives, and limiting relevance to some sectors.

### Interconnected Nature of Risks

AMR emerges and spreads through interconnected pathways linking animals, humans, and the environment. However, since it is beyond a single program's capacities to address all these pathways and drivers, many of these remain unaddressed programmatically [32]. While AMR data from clinical and hospital settings provide an important surveillance signal for humans, they capture only a fraction of the wider system in which resistance emerges and spreads. Given the extensive use of antimicrobials in livestock and the role of environmental reservoirs, understanding the role of antimicrobial use (AMU) and AMR transmission requires approaches tailored to animal and environmental systems rather than extensions of human health surveillance models.

### Anthropocentricity and Unequal Ownership

The Fleming Fund was funded and led from the human health sector, reflecting the clear burden of AMR on human health. While recognition of contributions from animal and environmental sectors motivated the adoption of One Health, in practice, animal and environmental components were often positioned in relation to a core human health program, rather than being developed as equally foundational elements in their own right [28, 33]. This had implications for sectoral ownership and long-term commitment. Objectives that mattered to animal and environmental health sectors were not sufficiently recognized or integrated, leading to difficulties in aligning sector interests, sustaining incentives for participation, and ensuring sustainability [34, 35]. This positioning also increased the vulnerability of animal and environmental components to budget reductions, particularly under wider fiscal constraints. These experiences highlight the importance of designing One Health programs with explicit mechanisms to support equitable ownership, resourcing, and priority-setting across sectors from the outset.

### Environment, AMR, and Conceptual Ambiguity

Important lessons about the conceptualization of environment can also be identified. AMR risks associated with the environment relate less to “environmental health” per se and more to the environment's role as a reservoir, mixing space and transmission pathway for resistance. Resistance genes can emerge and spread in environmental microbiomes due to antimicrobial residues from agriculture, aquaculture, pharmaceutical production, and human waste [36]. Because much of the One Health literature frames the environment primarily in terms of “environmental health,” it can obscure the role of environmental systems as reservoirs and transmission pathways directly linked to animal and human AMR outcomes. This framing can lead to deprioritisation of environmental activities, reinforced by limited technical capacity and chronic underfunding, constraining meaningful integration.

### Misaligned Surveillance Approaches and Stewardship

In human health, passive surveillance of hospitalized patients, while clinically appropriate, produced data biased toward hospital-acquired infections and poorly suited to assessing resistance patterns in the wider community.

In animal health, active surveillance was initially carried out in healthy chickens to estimate resistance in priority zoonotic bacteria to antimicrobials specified by the WHO as critical for use in humans, with additional species included over time [37]. Although intended to enable generation of comparable data, these data had no diagnostic relevance for veterinarians and farmers, reducing their immediate utility for stewardship and action with respect to antimicrobials used to treat disease in animals.

Alignment with human health priorities also led to risks in stewardship. In settings with limited veterinary epidemiology capacities, surveillance showing low or no resistance to medically important antimicrobials authorized for use in humans only (eg carbapenems) can unintentionally shift use away from lower-priority antimicrobials. In some contexts, this risked a potential escalation toward the use of critically important drugs in animal production systems, running counter to One Health stewardship objectives. It also underlined the importance of analytic capacity in all sectors as an essential ingredient to an effective surveillance system.

The Global Tricycle Surveillance protocol is an example of an integrated surveillance approach, collecting data on a single organism from humans, the food chain, and the environment, and was implemented in a number of Fleming Fund-supported countries (see Box 1) [38]. Further guidance on implementing integrated surveillance for AMR and AMU has recently been released by the Quadripartite [39]. The Fleming Fund experience supports the position in the guidance regarding the importance of strong sector-specific surveillance, with a need to start with sectoral strengthening. The Fleming Fund experience, therefore, points to the need for surveillance models that serve all sectors.

### Implications for One Health Design

Overall, the Fleming Fund experience demonstrates that effective One Health AMR programs require more than coordination and data sharing across sectors. Strong multisectoral action depends on strong individual sectors, with clearly articulated objectives, appropriate surveillance models, and capacity to interpret and communicate data for different audiences. Programs that center human health objectives without adequately accounting for sector-specific priorities and incentives, mandates, and resource constraints risk producing unintended and counterproductive outcomes. Efforts to develop shared resourcing could be one approach to addressing capacity constraints in an equitable manner (see Box 4).

Box 4. **Case Study 4: Shared Resourcing for AMR Surveillance**

In resource-limited settings, shared resourcing across human, animal, and environmental sectors has emerged as a pragmatic strategy for strengthening AMR surveillance and is often cited as an advantage of adopting a One Health approach [53]. Drawing on examples from Timor-Leste and Ghana, this section illustrates how leveraging existing sectoral strengths through shared laboratory infrastructure can advance multisectoral AMR surveillance.

At the start of the Fleming Fund Country Grant in Timor-Leste, antimicrobial susceptibility testing (AST) capacity was absent in the animal health sector [54]. Expertise and resources from public health labs were used to help establish AST capacity at the Veterinary Diagnostic Laboratory [55]. Animal-origin bacterial isolates were subsequently analysed through the National Health Laboratory, generating resistance profiles relevant to public health while reducing duplication of expensive infrastructure [54]. This approach improved efficiency, supported cross-sector learning, and helped harmonize laboratory methods across sectors [56].

Similar forms of shared resourcing have been reported in other Fleming Fund-supported countries, including Bhutan, Ghana, Laos, Nepal, Papua New Guinea, Rwanda, Uganda, and Vietnam. In these settings, national human health AMR reference laboratories supported other sectors through sample testing, isolate storage, equipment maintenance, and external quality assurance.

Ghana represents a more institutionalized model of shared resourcing. Multisectoral surveillance guidance shared technical working groups and a national digital dashboard integrate AMR, AMU, and AMC data across human, animal, and environmental sectors. The human health national AMR reference laboratory provides external quality assurance, while the Noguchi Memorial Institute for Medical Research serves as a shared genomic sequencing hub.

However, shared resourcing is not a substitute for sustained sectoral investment. In Timor-Leste, testing of animal isolates in human laboratory remained ad hoc, posing risks to sustainability. Stronger One Health operationalization would require formal agreements, standard operating procedures around biosafety and biosecurity, accreditation standards, and routine workflows that embed shared resourcing within routine surveillance systems.

Effective One Health design therefore requires explicit integrated and multisectoral objectives together with sector-specific objectives, sustained investment in technical and institutional capacity, and governance arrangements that balance cross-sectoral integration with equity and sustainability across human, animal, food, agriculture, and environmental systems.

## CONCLUSIONS

To conclude, the Fleming Fund program provided a unique empirical vantage point for generating practical insights into the operationalization of One Health approaches across diverse geographic and programmatic settings. Its experience demonstrates that, although it is vital and well-suited for addressing complex challenges like AMR, operationalizing the approach remains inherently challenging. Converting the One Health theory into effective action requires navigating complexities that are shaped by a range of context-specific factors, including political dynamics, sectoral priorities, and local circumstances. These challenges underscore the importance of adapting One Health interventions to the realities and nuances of the environments in which they are implemented, ensuring their practical feasibility and local relevance.

A central conclusion is that One Health should be understood as an approach or lens, requiring empathy, inclusion, and an awareness of the interdependence of different sectors. Instead of grouping all non-human health activities into a One Health module, the One Health approach needs to be incorporated into all aspects of program design. In the context of an AMR program, this would mean that all sectors are equitably resourced from the beginning of a One Health program and that the One Health approach addresses the multiple objectives of AMR and/or AMU surveillance in the contributing sectors to understand AMR risks within and between sectors.

A related lesson from the Fleming Fund experience is that generation of surveillance data alone is insufficient. The value of One Health surveillance depends on the capacity to analyse, interpret, contextualize, and communicate data to different audiences. Where this capacity is weak, surveillance findings may be misinterpreted or misapplied, undermining stewardship goals.

Another consistent lesson concerns the need for investment beyond the human health sector. Functional One Health systems depend on sector-specific capacities, incentives, and priorities being recognized on their own terms. Without meaningful resourcing of animal health, aquaculture, and environmental institutions, multisector coordination risks becoming symbolic rather than substantive, reinforcing existing asymmetries in representation and ownership and risking perverse outcomes.

Finally, the Fund's experience shows that One Health cannot be reduced to formal governance structures or surveillance alone. The fellowship program and country experiences described in the paper illustrate that informal networks, peer learning, and professional relationships are equally critical to enabling and sustaining collaboration, building trust, and generating policy-relevant knowledge.
